# Patient perspectives on home-spirometry in interstitial lung disease: a qualitative co-designed study

**DOI:** 10.1136/bmjresp-2023-001837

**Published:** 2023-10-03

**Authors:** Jessica Mandizha, Joseph W Lanario, Anna Duckworth, Sarah Lines, Ana Paiva, Victoria Elworthy, Veena Muraleedharan, Ana Jorge Da Ponte, Rebecca Shuttleworth, Graham Brown, Howard Almond, Carole Bond, Maureen Cosby, Joanne Dallas, Marium Naqvi, Adam David Russell, Alex Berry, Michael Gibbons, Christopher J Scotton, Anne-Marie Russell

**Affiliations:** 1 Interstitial Lung Disease Unit, Royal Devon University Healthcare NHS Foundation Trust, Exeter, Devon, UK; 2 School of Medicine, University of Plymouth, Plymouth, UK; 3 Clinical & Biomedical Science, University of Exeter, Exeter, Devon, UK; 4 EPIC Group, University of Exeter, Exeter, Devon, UK; 5 Respiratory Medicine, Guy's and St Thomas' NHS Foundation Trust, London, UK; 6 Creative Arts, AR Design Studio, Bristol, Somerset, UK; 7 Academy of Nursing, University of Exeter, Exeter, Devon, UK; 8 Medical School, University of Exeter, Exeter, Devon, UK

**Keywords:** interstitial fibrosis, respiratory function test, patient outcome assessment

## Abstract

**Background:**

Opportunities for home-monitoring are increasing exponentially. Home- spirometry is reproducible and reliable in interstitial lung disease (ILD), yet patients’ experiences are not reported. Given the morbidity and mortality associated with ILDs, maintaining health-related quality-of-life is vital. We report our findings from a codesigned, qualitative study capturing the perspectives and experiences of patients using home-spirometry in a UK regional ILD National Health Service England (NHSE) commissioned service.

**Methods:**

Patients eligible for home-spirometry as routine clinical care, able to give consent and able to access a smart phone were invited to participate. In-depth, semistructured interviews were conducted at serial time points (baseline, 1, 3 and 6 months), recorded, transcribed and analysed thematically.

**Results:**

We report on the experiences of 10 recruited patients (8 males; median age 66 years, range 50–82 years; 7 diagnosed with idiopathic pulmonary fibrosis, 3 other ILDs) who generally found spirometry convenient and easy to use, but their relationships with forced vital capacity results were complex. Main themes emerging were: (1) anticipated benefits—to identify change, trigger action and aid understanding of condition; (2) needs—clinical oversight and feedback, understanding of results, ownership, need for data and a need ‘to know’; (3) emotional impact—worry, reassurance, ambivalence/conflicting feelings, reminder of health issues, indifference; (4) ease of home-spirometry—simplicity, convenience and (5) difficulties with home-spirometry—technical issues, technique, physical effort.

**Conclusion:**

Home-spirometry has many benefits, but in view of the potential risks to psychological well-being, must be considered on an individual basis. Informed consent and decision-making are essential and should be ongoing, acknowledging potential limitations as well as benefits. Healthcare support is vital.

**WHAT IS ALREADY KNOWN ON THIS TOPIC**

- We know that home-spirometry in interstitial lung disease (ILD) is feasible, reliable and acceptable, however, patient experience of home-spirometry has not previously been reported.

**WHAT THIS STUDY ADDS**

- Undertaking home-spirometry for patients with ILD can be a positive experience but may pose risks to psychological well-being.

**HOW THIS STUDY MIGHT AFFECT RESEARCH, PRACTICE OR POLICY**

- When undertaking home-spirometry, informed consent is essential and should be ongoing. Care must be individualised and quality of life prioritised. Ongoing healthcare support is vital.

## Introduction

Interstitial lung diseases (ILDs) are a diverse group of respiratory diseases characterised by inflammation and scarring of the lung tissue. Lung damage is often irreversible, progressive and morbidity and mortality rates are high.^1^ Symptoms include breathlessness, cough and fatigue.^2^ Treatment for ILD focuses on relieving symptoms, improving or maintaining quality of life (QoL) and slowing disease progression.

Forced vital capacity (FVC) is an accepted clinical marker of this disease progression.^3^ Pulmonary function tests pre-COVID-19 conditions were performed in the clinic at 3–12-monthly intervals, supplemented by ad-hoc home-spirometry to capture FVC. Post-COVID-19, handheld home-spirometry devices are more available and affordable and opportunities for remote monitoring programmes to be embedded in clinical care are increasing.^4–9^

The first study using home-spirometry for patients with ILD was published in 2016^10^ with subsequent feasibility and acceptability repeatedly demonstrated.^11–15^ Reliability and reproducibility of home-spirometry devices are confirmed by a systematic review^16^ and an observational study highlights positive patient experiences with the majority wishing to continue with home-spirometry after a 6-week programme.^17^ Yet, in ILD, where maintaining or improving QoL is a key treatment aim, little is known about the impact that engaging with home-spirometry has on health-related quality-of-life (HRQoL) for patients.

To understand patients’ lived experience, in-depth inquiry is needed. Qualitative research provides rich, nuanced and contextualised data, which allow for the complexity of patient experiences and will inform high-quality, patient-centred care.^18 19^

### Aims

1. Characterise patient understanding and expectations of home-spirometry.
2. Capture in-depth patient experiences of using handheld spirometers.
3. Develop preliminary recommendations to optimise supported self-management in ILD.

## Methods

### Study design

#### Patient and public involvement

Exeter Patients in Collaboration for Pulmonary Fibrosis Research (EPIC-PF, n=12) informed the design of this exploratory, qualitative study. As users of digital devices, they determined a need to examine the experiences of patients with ILD undertaking home-spirometry. EPIC-PF members participated in two focus groups to elicit the question guide with final questions agreed by consensus. Patient partners reviewed interview transcripts and actively contributed to the writing of this manuscript.

In-depth, semi-structured interviews were conducted with study participants at serial time points (baseline, 1, 3 and 6 months).

### Recruitment and selection

Patients eligible for home-spirometry as routine clinical care attending a regional National Health Service England (NHSE) specialist commissioned ILD service in the UK, who were able to give consent and had access to a smart phone were included. Patients were excluded if a contraindication for spirometry was present. A purposive sampling approach captured a range of diagnoses, age groups and baseline lung function.

### Home-spirometry programme

Patients were provided with a MIR 'Spirobank Smart’ spirometer. Training and technical support were provided by a respiratory physiologist and nurses. Technique was reviewed 3 monthly or as needed. Patients were asked to perform one acceptable (parameters set within the app) manoeuvre on a weekly basis and submit their weekly result in PDF form to the ILD nursing team via email. Results were minimally ‘routinely’ checked monthly, and ad-hoc in response to patient-initiated contact about their readings. Clinical concerns were discussed with specialist ILD clinicians.

### Data generation

Interviews were conducted via online conferencing software by JM and second interviewer (A-MR/JWL). Preformed, open-ended questions guided discussion, with other topics explored as they emerged (see online supplemental material for interview guide). Twenty-eight interviews were completed, lasting 15–60 min.

10.1136/bmjresp-2023-001837.supp1 Supplementary data

### Analysis

Interview transcripts generated by conferencing software were cross-checked with the original recordings, edited for accuracy, consistency and anonymised. Transcripts were read repeatedly for familiarity, with emergent patterns identified inductively; themes and subthemes were generated. NVivo qualitative data analysis software, V.1.6.1 (QSR International (UK) Limited) supported coding labels for the data. Data, coded independently by JM and JL, were scrutinised, compared, discussed and ratified by AMR and patient participants. Emerging themes were discussed with the ILD clinical team.

### Ethical considerations

The project was registered, peer-reviewed and approved as a service user involvement project (Ref: 20–4946) by an NHS Respiratory Specialty Governance Group. Written, informed consent to participate in the study was obtained in line with National Institute for Health Research good clinical practice guidelines.

## Results

Ten patients agreed to participate. Nine were interviewed at baseline, four at 1 month, seven at 3 months and eight at 6 months. Patient demographics are reported in table 1.

**Table 1:** Patient demographics

Patient	Gender	Age range (years)	Diagnosis	Baseline FVC (L/% predicted)	Baseline DL_CO_ (mmol/min/kPa/ % predicted)	GAP score
1	M	60–69	CTD-ILD	2.45 L/61	3.35/41	N/A
2	M	70–79	IPF	3.01 L/74	3.52/42	5 Stage II IPF
3	M	50–59	DI-ILD	2.79 L/66	4.71/49	N/A
4	M	60–69	IPF	3.36 L/102	5.83/67	3 Stage I IPF
5	F	70–79	IPF	2.24 L/81	2.61/40	3 Stage I IPF
6	F	50–59	CTD-ILD	2.66 L/80	3.60/53	N/A
7	M	60–69	IPF	2.02 L/48	2.28/27	7 Stage III IPF
8	M	60–69	IPF	3.85 L/91	5.51/64	2 Stage I IPF
9	M	70–79	IPF	2.49 L/67	Not available	
10	M	80–89	IPF	3.44 L/87	4.46/54	4 Stage II IPF

CTD-ILD, connective tissue disease-interstitial lung disease; DI-ILD, drug-induced-interstitial lung disease; IPF, idiopathic pulmonary fibrosis.

Six patients stopped using their home-spirometry devices between 3 and 6 months. Reasons for stopping were cited as anxiety (n=2), cough (n=1), chest pain (n=1), physical deterioration (n=1) and overseas travel (n=1). The four remaining patients switched to an alternative app-based system. Beyond the parameters of the study, two patients continue to perform spirometry weekly and two patients have reduced to monthly testing after finding weekly measurements intrusive. Eight patients attended the closing-out 6-month interview enabling the opinions of those no longer using devices and their reasons to be captured in the results.

Five key themes characterise patients’ experiences of home-spirometry: ‘Anticipated Benefits’, ‘Needs’, ‘Emotional Impact’, ‘Ease of Home-spirometry’ and ‘Difficulties with Home-spirometry’. The themes and associated subthemes are discussed here, alongside illustrative quotes (tables 2–6).

**Table 2:** Theme 1: anticipated benefits (patient number, time point of interview)

Subtheme	Illustrative quotation
Identifying change	*I want to obviously make sure that if there is, there any change that it’s recognized as soon as possible. (4, BL*) *…basically, from my position, anything that will help in assessing my, if you like, overall condition and whether it’s deteriorating, or whether it stabilized or whatever, anything that I think that can help to support that, I’m glad to support myself. (7, BL*)
Triggering action	*…as I understand it, if there’s a rapidly deteriorating trend…hopefully it will trigger a conversation and maybe some action of some sort. (8, BL*) *…if it was that way, I'd be very breathless, and I would be thinking I need help anyway. But the spiro would, as it has done so far, say to me “You are as bad as you feel. You need to do something.” (3, 3m*)
Understanding condition	…*it’s important to know your enemy, as they say in the military, and also how you can control your condition. So, I think using spirometers can help with that. (3, BL*) *…all I'm trying to do is understand my condition, which is why I was so happy when this offer of home-spirometry came along and try to understand more, to, to be able to measure it…in order to plan my life…I need to know what’s happening. (2, 3m*) *I suppose it’s having a more thorough understanding of my level of, if you like, decline or if I’m steadying out over a period of time, just to know what to expect around the corner, really… (7, 6m*)

BL, base line; 3m, 3 months; 6m, 6 months.

**Table 3:** Theme 2: needs (patient number, time point of interview)

Subtheme	Illustrative quote
Need for clinical oversight	*You know, it’s the feeling that somebody will check those data. You know over a period of time, somebody’s keeping an eye on it. So, it’s not four to six months between people knowing how you are. (1, 3m*) *Yes, I mean, just because I’m doing it, I’ll still get all the support from the hospital, won’t I? (5, BL*) *But if I know that you're reviewing that…then that might actually help quite a lot with me not focusing on little changes, but just being reassured that you're looking at it, and if there is anything you're gonna pick it up and I don't need to monitor it myself. (6, BL*)
Need for feedback	*You'd sent me a message and said, yeah, I'm glad to see that return to baseline. And you showed no concern. That was actually quite good. I got that after the drop, so I was already feeling anxious, you know? Yeah, but it was nice to get that little bit of feedback. (1, 3m*) *So I'm sort of trying to really get a sense of what it actually means in terms of disease progression, so I can only really get that in a discussion. (8, 1m*)
Need for understanding	*I don't fully appreciate and understand that report and I’d like to understand how it’s read properly, but, most importantly, how I can compare that with previous reports. (7, 3m*) *…you get the data and are then trying to understand. If I don’t have the understanding to come back to it, I’m worse off than not knowing, almost. (8, BL*)
Need for ownership	*I think it’s nice to chart progress and hold records. Will be a very simple record on my phone in this case, but before I've been holding them written down. (3, BL*) *But I still log it on my own chart, you know? I've got a little spreadsheet. You have to keep track of things. (1, 3m*)
Need for data	*I think…about…I think about all those data points…they’re covering quite a bit of a span. My mind naturally thinks you want to measure something that is deteriorating on a far more regular basis. It is deeply ingrained in my mind and professional behaviour. (8, BL*) *Okay, so I’m doing all of those things in terms of my numbers, if you like. So, my weight, my sat levels, my spiro scores, my pulse rate, my resting pulse rate and I’m recording all of those, number of steps I do in a day, the number of miles I cycle. (3, 6m*)
Need ‘to know’	*As I said, I want to know exactly what is happening. My lifespan is limited, and you can almost see the horizon looming in front of you very quickly. I want to know basically how quickly this condition…if it is progressing at all and how quickly, so that I can adjust my lifestyle. (2, 1m*) *It’s nice that somebody can tell you what the true picture is, whether it’s good or bad. It’s still important, I think… (1, 3m*)

BL, base line; 1m, 1 month; 3m, 3 months; 6m, 6 months.

**Table 4:** Theme 3: emotional impact (patient number, time point of interview)

Subtheme	Illustrative quote
Worry	*…it just sounds like the clock is ticking. It nibbles away and puts a worm in my mind, and I see the decline. I see it in front of me and as you said it is a hard fact and it is not a pleasant experience for me, unfortunately. It worries me. It causes me anxiety that I could probably do without. (1, 6m*) *‘there’s just a quiet, simmering anxiety and I look at the box now as it’s sitting beside me with the spirometer in and it’s a bit like thinking you know- you’re gonna get me. You’re gonna disappoint me’ (1, 3m*) *To be quite truthful, I really don’t want to know what’s going on…I know it’s going downhill, I just don’t really want to know. (Patient 9, 6m*) *Well, it’s, it’s not, it’s not a pleasant thing, physically. It’s not a horrible, but it’s…it’s also you can, at the back of your mind, you’ve got this, sort of, nagging feeling that you might get a bad result. And you’re starting to degrade. (8, 1m*)
Reassurance	*It is useful as in general reassurance that things are not getting that much worse. (4, 1m*) *I think the next test will give me an idea if things have improved again. I want to see that dial thing going upwards and not down because I will know then if these tablets are going to work. (9, 3m*)
Ambivalence/ conflicting feelings	*I’m slightly worried that I might obsess a little bit over it…It might be reassuring for me. It might also be the opposite. (6, BL*) *It’s a bit like opening a present- you don't know whether you're going to be disappointed or delighted. (1, 3m*)
Reminder	…*It makes me think of something I try not to think about…bottom line, it’s physically unpleasant and it makes me realise this is, is a pretty serious thing, rather than something I can, kind of, avoid. (8, 1m*) *…inevitably, I'm going to think about it more when I have to do home-spirometry every week, because at the moment, I might go for weeks and weeks when I don't think that I've got lung condition and spirometry will probably bring it more to the fore. (6, BL*)
Indifference	*Um, I suppose, because it’s very much part of my routine. I suppose the only thought… I wouldn’t be stressing about, you know…It doesn't make me feel “oh good it’s spiro day” I feel like it’s part of routine. (3, 3m*) *JM: And do you think it’s impacted your well-being? No. Not at all…neutral, I’d say. Yeah. (7, 6m*)

BL, base line; 1m, 1 month; 3m, 3 months; 6m, 6 months.

**Table 5:** Theme 4: ease of home-spirometry (patient number, time point of interview)

Subtheme	Illustrative quote
Simplicity	*I think in practical terms in some ways it exceeded my expectations because the device itself is compact, easy to use, easy to take away if you are going somewhere. (1, 6m*) *I think it’s very simple to clean. It’s very simple to use. The…software is…very easy to use, as well. Yeah…I don't think I have any other issues. (3, BL*)
Convenience	*It just only takes, you know, 10 minutes at most, no traveling involved, no waiting…I think it’s much more relaxed and much easier to do. (6, 6m*) *…especially when you have had nearly 2 hours to get to hospital and then go through that…now we haven’t got the car. A round trip on the bus can take up to 5 hours. (9, 3m*) *…it saves me a 60-mile round trip and half a day driving round the hospital trying to find a parking space…it’s a nightmare. (1, 3m*)

BL, base line; 3m, 3 months; 6m, 6 months.

**Table 6:** Theme 5: difficulties with home-spirometry (patient number, time point of interview)

Subtheme	Illustrative quote
Technical issues	*Well, in the beginning difficult, purely because of the lack of knowledge on the internet, but when my daughter downloaded the app to the phone she knew how to use it straight away. Someone like me who has only a basic knowledge of the internet struggled. If it was somebody using it that had no knowledge at all…they just would not be able to cope with it without backup. (9, 3m*) *My new phone is fine but we haven’t been able to complete the test result yet. I tried to do that after we had our discussion at 10 o’clock. Got all the bits and pieces in place and tried to do this but didn’t complete it so there is probably something we need to go through again. (10, BL*)
Technique	*I think it’s still, it’s a slightly fiddly thing to do in terms of, you know, I get as many…failed tests, as I do successful tests. I just end up blowing maybe four times to get results, so there’s that about it, but that is more of a sort of minor irritation than a problem. (8, 3m*) *I was actually getting quite upset with it at one point where, you know, sort of, it took me six goes to get three acceptable trials, what have I done differently there that you think is not right? (4, 3m*)
Physical effort	*I’ve had a horrendous cough and a lot, a lot of chest infections. I haven’t felt that I can actually cope with a lot more than the cough and the chest infections, because if I’m honest, I felt like rubbish. (5, 6m*) *The only thing is, it does drain you a little bit when you do the test but that is a short-term issue. (10, BL*)

BL, base line; 3m, 3 months; 6m, 6 months.

### Theme 1: anticipated benefits

Patients were able to identify potential benefits of regularly performing lung function tests at home. Importantly, these are different to realised benefits, with patients only predicting the positive impact of home-spirometry (table 2). Three subthemes are identified:

#### Identifying change

Patients anticipated that using home-spirometry would help identify decline in lung function quickly or capture helpful trends, such as improvement following a change in treatment. Identifying change in a timely way was deemed important, rather than waiting for periodic hospital-based measures. Several referenced difficulties in accessing routine lung function tests and follow-up appointments were imposed by the COVID-19 pandemic.

#### Triggering action

Patients anticipated that the programme would potentially trigger actions which may not have occurred otherwise. These included patient actions, such as contacting their clinician or adjusting their lifestyle; or clinician actions, for example, ordering further investigations, instigating or changing treatment. Triggering a timely conversation with the clinical team was identified as having value, even in the absence of changes in care or improvements in symptoms.

#### Understanding their condition

Patients felt home-spirometry may help to ground them in reality or assist with understanding their prognosis. Several expressed a hope that the information they gained would help them make significant life decisions. For some patients, this increased understanding potentially offered reassurance if their results were stable.

### Theme 2: needs

To benefit fully from the home-spirometry programme, patients described their requirements of the clinical service, expressing underlying needs, which may have influenced their decision to undertake home-monitoring (table 3). Six subthemes are identified:

#### Need for clinical oversight

Patients stated a need for the results to be reviewed regularly by the clinical team. Some were content to submit their results without scrutiny, for clinicians to interpret.

I won't worry about it. I'll leave it all to you lot to worry about. I'm sure you'll come back and tell me if it’s good or bad, won’t you? (5, BL)

Others made it clear that they did not want any change in their standard care, for example, less face-to-face contact and it was essential for patients to have a point of contact to direct concerns towards (table 3).

#### Need for feedback

Patients needed information about the results to be passed back to them in a predictable and consistent way, that is, via email, phone call or a face-to-face consultation. They recognised this feedback as a source of reassurance and encouragement, regardless of whether the results had declined or not.

If it has had some effect on attitude, it’s mainly psychological. Social psychology. Just to know that somebody out there is interested, it’s good. (2, 6m)

#### Need for understanding

Patients needed an in-depth understanding of the spirometry report, the clinical interpretation of this and the implications for their own health. Having the ability to compare their current results with previous results was especially important. Some patients expressed concern around misinterpreting the results and not understanding the results fully was a source of anxiety.

#### Need for ownership

Patients expressed a need to possess, use and share their own information regarding their health. This included collating their results on spreadsheets, graph paper or diaries. Some referred to sharing the results with their respiratory physician or interested family members. Some patients expressed frustration at not having easy access to hospital results.

I think it is a handy little machine, so the person using it can keep an eye on what is happening themselves. I mean, when you go to the hospital, they don’t tell you how well you are doing there or how well you are not doing. They have to wait for someone else to write to you and tell you. (9, 3m)

#### Need for data

Several patients indicated that they value data and wanted additional empirical information about their health condition. Having more information to discuss or share was seen as a clear benefit to home-spirometry. This was most apparent in those with a professional background in science or engineering (n=3).

I'm quite scientific in my thinking, so data is great. (6, BL)

#### Need ‘To know’

Patients expressed a need for knowledge around their condition, particularly in the context of their prognosis. One patient described this as gaining a ‘true picture—good or bad’ and found not knowing if they were declining a source of anxiety. They felt that the additional lung function results, in some way, would prepare them mentally if they declined.

Patients described the practical impact this knowledge would have on their lives. For example, one patient was trying to decide whether to travel immediately or delay the trip. Another felt the additional information would help them make decisions about how quickly they needed to reorganise their home.

My mind-set is, you know, then you can make the most of what you've got in a more sensible way and that’s very different if it’s a long draw- out process, than if it’s kind of rapid…and the more I can know about where I am, conceivably, in all instances, the better I will feel. (8, 1m)

### Theme 3: emotional impact

Patients expressed positive and negative emotions triggered by taking part in the home-spirometry programme, performing spirometry or viewing results (table 4). Five subthemes are identified:

#### Worry

Worry was significant across all patients and time points, frequently relating to a decline in FVC and the implications of this for their disease trajectory. Worsening results, even when not considered clinically significant or linked to worsening symptoms could elicit stress.

When I have the odd bad day, I generally don’t worry about it as much. I think tomorrow will be better and usually it is and get back to doing what I normal do. The numbers don’t have to vary much for me get anxious. (1, 6m)

This patient described feeling nervous and hesitant to repeat the spirometry after a lower result, which generated a constant, low-level feeling of anxiety which peaked at the point of testing. Following a drop in FVC, he started to feel unwell physically, uncertain whether this was a genuine change in symptoms or psychosomatic.

Worry was not always based on actual results, but triggered by an anticipation of poor results, so even something as simple as looking at the spirometer box would be enough to generate anxiety. A further source of worry for some patients stemmed from not feeling that they understood the result fully or their implications (table 4).

One patient coped with this by reminding himself that it is normal to have some variation in data and it does not necessarily indicate a permanent or irreversible decline. Others responded to worry about changing symptoms or FVC results by avoidance.

So, because of that ‘up and down’ I just…have made the decision to try not to think too much about it. (6, BL)

I’m not burying my head in the sand about all this, but what’s the point? I know if I…so really, what is the point? (5, 6m)

#### Reassurance

In contrast to this, patients sometimes found that using their home spirometer helped reduce their fears. For some patients, seeing a steady result and knowing that their condition was not worsening was reassuring. Even if they were feeling well, it was important to have this corroborated by their spirometry results.

But then, when it’s consistent, hasn't changed, then it makes you feel better the rest of the week. (8, 1m)

For others, not knowing where they were on their disease trajectory was a source of anxiety and the spirometry results were in some way reassuring, even if they demonstrated a decline.

Some found their results reassuring if they improved as they recovered from an exacerbation or increased their exercise, or if their results were stable while taking antifibrotic medication. Others, regardless of their results, found reassurance in having clinical oversight or simply felt reassured by the proactive nature of the programme.

It is useful because it’s reassuring to know that something positive is happening, rather than leading a negative life trying to ignore my condition. (3, 3m)

#### Ambivalence/conflicting feelings

Patients frequently experienced simultaneous conflicting reactions, beliefs or feelings about using home-spirometry, containing both positive and negative components. Some expressed feeling uncertain about how they might respond to home-spirometry, particularly if their results showed a decline.

…if I have any problems, and I will definitely, you know, ring or speak to whoever it is and say, look, I don't feel I can do this anymore because it’s getting me down. Yeah. It shouldn’t do, should it? (5, BL)

Others were concerned that their results might affect how they feel, rather than how they feel affect the results, or that they might become hypersensitive to minor changes and found this unsettling.

Other patients discussed the tension between finding the results worrying or reassuring and the desire to know whether their disease was progressing while simultaneously wanting to avoid this reality.

It’s a once weekly chore, that has the potential to make me feel less concerned about deviation and has the hidden…the possible fear that… when I've got to do the readings that they might have gone down and that might mean something. (8, 1m)

There was a tension between finding the results motivational, if there was a positive response to treatment or exercise, and disempowering, if limited action could be taken following declining results.

#### Reminder

Using the home spirometer acted as an unpleasant trigger or undesirable reminder of health issues. Several patients felt that they could otherwise go for many weeks without thinking about their lung condition, but home-spirometry bought this to the fore. The fact that spirometry is a physical act increased its power as an ‘unpleasant reminder’ of a serious condition. One patient felt this undermined what he considered to be a coping strategy:

For many years, since I was diagnosed my reaction to this illness was put it to one side. I saw Dr G every 4, 5 or 6 months, did my PFTs, and got on with life, head down…I think that is probably my way of dealing with it and having something that reminds me every week or periodically, it doesn’t help. (1, 6m)

A further patient found weekly measurements intrusive, he was preoccupied with the spirometry for much of the day when he performed his tests. He therefore decided to reduce the frequency of testing to once a fortnight.

#### Indifference

For some patients, anticipating using the spirometer, performing spirometry or viewing the results caused neither a positive nor a negative emotional reaction. This was simply ‘part of their routine’ and was a source of neither stress nor enjoyment. Three patients anticipated at baseline that using home-spirometry would not impact their well-being. Two reiterated this at their final interview. The third patient admitted that he found observing his decline troubling and that he would ‘rather not know’.

### Theme 4: Ease of home-spirometry

Patients described how comfortable they were using the spirometry device (table 5). Two subthemes are identified:

#### Simplicity

Patients described an ease with downloading the software, connecting the device to the app, forwarding results and performing spirometry. Cleaning and caring for the device was ‘straightforward’. The number of references made to ‘ease’ increased over the 6-month period.

#### Convenience

Performing home-spirometry fitted into patient’s schedule with minimal inconvenience. They found the process took less time and energy than hospital-based tests, especially important for those with more physical limitations. They also appreciated that there was no travelling, waiting time or parking issues. For patients living rurally or reliant on public transport, the time and effort saved were significant.

### Theme 5: Difficulties with home-spirometry

Patients described problems they experienced using the spirometry device (table 6). Three sub-themes are identified:

#### Technical issues

These included difficulty downloading the software, connecting the device to the app or forwarding results. Several patients made the decision to update their phones to join the home-spirometry programme. References to technical issues decreased over time, with no issues raised at the 6-month time point.

#### Technique

Patients expressed difficulty with the act of performing spirometry. This included timing the blows, for example, avoiding a hesitant start; performing blows, for example, avoiding an abrupt stop or achieving a consistent result each session or over a longer period of time. References to issues with technique peaked at 1 month, then reduced over time until no references were made at the 6-month time point.

#### Physical effort

Many patients described how physically challenging performing spirometry can be. It might result in unpleasant symptoms such as light-headedness or cough or leave the patients feeling drained. This was significantly worse when the patients had a chest infection or viral illness. One patient, with CTD-ILD, found she struggled accessing, assembling and cleaning the spirometer when experiencing a flare due to pain in her hands.

The frequency of patients referencing the physical effort of home-spirometry was consistent over the 6-month period. At the 6-month time point, many patients described particular difficulties in using the spirometer when unwell, avoiding it as a result.

## Discussion

This study uniquely captures the expectations and experiences of patients with ILD using home-spirometry devices. Previous observational studies of home-spirometry for patients with idiopathic pulmonary fibrosis (IPF) reported positive experiences such as devices being ‘useful and empowering’ over a 4-week period^11 17^ and wanting to continue to use home-spirometry devices after 6 weeks.^17^ Here, anticipated benefits of home-spirometry, including capturing change and triggering action reflect those of the clinical team, with the additional anticipated benefit of contributing to patients’ insight into the nature of their condition. These anticipated benefits were not always realised and despite the relative ease of processes, the perceived magnitude of such benefits may not always be sufficient to motivate patients to continue. Patients expressed frustration when a decline was noted but limited action could be taken, or when their FVC dropped and they simultaneously became symptomatic, not offering the ‘early warning’ they hoped for. Honest conversations with patients at the point of consent are an ethical imperative to manage patient expectations and specifically address what actions may or may not be possible in the event of lung function decline.

Patients expressed personal needs that also provided motivation for undertaking home-spirometry, for example, a need for ownership of their own results. They also expressed needs relating to the role of the clinical team, for example, the need for oversight and feedback. This feedback was important to patients even when their results were stable and their symptoms unchanged. Discussions with patients at the point of consent and reaching an agreement on the nature and level of contact they need will help to manage expectations and reduce anxiety. Having a point of contact to discuss technical problems as well as clinical concerns is vital. Previous studies have highlighted this.^13^ This will influence the workload of the clinical team and demonstrates a need for the development of digital care pathways which formalise monitoring and support systems. Health economic evaluation of home-spirometry is required to accurately gauge the health resource implications.

While evidence from a non-blinded randomised controlled trial found home-monitoring did not improve overall HRQoL as measured by the King’s Brief Interstitial Lung Disease Questionnaire (K-BILD) excepting psychological well-being,^13^ our results clearly indicate positive and negative emotional impact of undertaking home-spirometry. While reassuring and empowering for some, others found it a source of significant psychological distress, an issue raised previously.^10^ It is important that the potential for distress is discussed at the point of consent and that patients explicitly understand that taking part is optional. Feelings may change over time and such discussions must be revisited periodically. Peer support mechanisms may be useful and relieve pressures on clinical teams.

Patients frequently raised the need to understand the results fully, despite initial training and provision of written information. The home-spirometry system used involved patients receiving a full pulmonary function report, with metrics not clinically relevant to their condition such as peak expiratory flow rate, challenging interpretation. Post-study, this was addressed by switching to an alternative app, which provides patients with just the FVC and FEV_1_ (forced expiratory volume in one second). Further work is needed to develop appropriate patient-centred resources.

Although the process was physically demanding, patients generally found the technology easy to use and convenient. As patients consistently avoided home-spirometry when feeling unwell, absence of data for a certain period could be used as a trigger to initiate contact with patients.

Patient preferences for spirometry testing were gathered via an international online survey^20^ from 760 respondents and reports some parallel findings. Patients wanted to be able to understand their results and what they meant for them. They wanted access to their results and the ability to compare them. The majority reported that the difficulty of performing spirometry was acceptable to them, although a minority (17%) did find it difficult. This was especially so for respondents with IPF (26.7% responding ‘somewhat acceptable’ or ‘not at all acceptable’). Finally, the survey reported that, for a small proportion of respondents, spirometry is ‘very worrisome’ or ‘causes extreme anxiety’.

In considering the psychological impact for patients, it is important to understand that avoidance represents a vulnerability factor in adjusting to chronic disease and if this is recognised it may present an opportunity to provide the emotional and informational support required to assist patients in adjusting to their diagnosis.^21^ Patients valued conversations with their clinical team, even if they did not result in a change in treatment or improved outcome. Declining results may provide a timely prompt to sensitively discuss end-of-life care.

These results support the existing evidence that there is a place for home-monitoring in the care of patients with ILD,^22 23^ but it must always be considered on an individual basis, especially in view of the potential risks to psychological well-being. Patients particularly valued participation in the final interview, which facilitated the transition back to the clinical care pathway and informed decision-making as to whether to continue home-spirometry. Further longitudinal work may lead to a better understanding of the predictive value and association between decline in symptoms and FVC. Informed consent is essential, including a discussion of potential limitations as well as benefits. The ongoing healthcare support required will have practical and economic implications, but fiscal implication may well be offset by providing high-quality, patient-centred home-monitoring services.

### Limitations

This is a small, single-centre study limiting our ability to draw conclusions on themes over time. Patients had a variety of ILD diagnoses. Patients, with CTD-ILD, experienced their spirometry results improving over time. This is not the natural course of IPF and may have given a more positive slant to the patient experience than might be expected with patients with IPF alone. The sample may be biased towards proactive patients who have the required technical skills. The home-monitoring service was set-up rapidly, out of necessity, during the COVID-19 pandemic. Patients may have been experiencing the isolation effects of lockdown. The home-spirometry system used involved patients receiving more detailed information than required, which may have caused confusion. Patients were required to submit reports in PDF format, via email, adding workload. This was also difficult and time consuming for clinicians, particularly to elicit changing values. Interviews were conducted by members of the patients’ care team, which may have impacted their ability to speak candidly.

### Recommendations

Further research should recruit patients from a wider demographic, including those in underserved communities who may experience digital poverty. Patients with a range of ILD diagnoses should be observed, to capture any differences between these groups. Patients with ILD are living with chronic conditions that will potentially require monitoring for many years. This study demonstrates that patients’ experience changes over a 6-month period, indicating the need for further longitudinal data. Finally, capturing the experience of other stakeholders, such as carers, the clinical team at the specialist centre and primary care staff are key to optimising the use of home-monitoring technology.

## Data Availability

Data are available upon reasonable request. Deidentified participant data, thematic analysis and coding system will be made available in response to reasonable request made to the corresponding author. We will seek approval from our governance team and patient research partners advisory board.
