# Supplementary material for: Patient perspectives on home-spirometry in interstitial lung disease: a qualitative co-designed study
Source: BMJ Open Respir Res. 2023 Oct 3;10(1):e001837. doi: 10.1136/bmjresp-2023-001837 (PMC10551997; doi:10.1136/bmjresp-2023-001837)
Supplement: Supplementary data [file bmjresp-2023-001837supp001.pdf]

bmjresp-2023-001837 Supplementary Interview Questions

Baseline Interview

- Tell me about your decision to undertake home spirometry?
- Do you anticipate any benefits?
- Do you anticipate any difficulties?
- How do you think home spirometry might affect your care?
- Do you think it will impact your wellbeing? In what ways?
- What do you understand by the term FVC? Has anyone ever explained to you?
- What's important to you in terms of monitoring your condition at home?

One month interview

- Describe your routine around using the spirometer (eg how many blows/time of day etc)
- How do you find the process?
- What do the results mean to you?
- Is it useful to you? In what ways?
- Do you think your readings reflect your physical symptoms?
- Tell me about your experience of home spirometry compared to hospital spirometry?
- What would be helpful for you, in terms of monitoring your own condition at home?

Three month interview

- Describe your routine around using the spirometer (eg how many blows/time of day etc)
- How do you find the process?
- What do the results mean to you?
- Is it useful to you? In what ways?
- Do you think your readings reflect your physical symptoms?
- Tell me about your experience of home spirometry compared to hospital spirometry?
- What would be helpful for you, in terms of monitoring your own condition at home?

Final interview

- How would you describe your experience of using home spirometry over the last few months?
- How do you think home spirometry affected your care?
- Do you think it impacted your wellbeing? In what ways?
- What would be helpful for you, in terms of monitoring your own condition at home?
- What's important to you in terms of home monitoring?
